# Effect of Hemoglobin, Albumin, Lymphocyte Count, and Platelet (HALP) Score on Survival of Patients with Metastatic Thyroid Cancer Treated with Tyrosine Kinase Inhibitors

**DOI:** 10.3390/jcm14041306

**Published:** 2025-02-16

**Authors:** Hikmet Öztop, Fazıl Çağrı Hunutlu, Selin İldemir Ekizoğlu, Özen Öz Gül, Soner Cander, Ahmet Bilgehan Şahin

**Affiliations:** 1 Department of Internal Medicine, Faculty of Medicine, Bursa Uludag University, Bursa 16059, Turkey; drselinildemir@gmail.com; 2 Division of Hematology, Department of Internal Medicine, Faculty of Medicine, Bursa Uludag University, Bursa 16059, Turkey; fazilhunutlu@gmail.com; 3 Division of Endocrinology, Department of Internal Medicine, Faculty of Medicine, Bursa Uludag University, Bursa 16059, Turkey; drozenoz@gmail.com (Ö.Ö.G.); scander@uludag.edu.tr (S.C.); 4 Division of Oncology, Department of Internal Medicine, Faculty of Medicine, Bursa Uludag University, Bursa 16059, Turkey; absahin@uludag.edu.tr

**Keywords:** HALP score, thyroid cancer, tyrosine kinase inhibitors, survival, nutrition

## Abstract

Tyrosine kinase inhibitors (TKIs) are crucial for improving the survival rates of individuals with metastatic thyroid cancer. Moreover, systemic inflammation and malnutrition are known to negatively affect metastatic thyroid cancer prognosis. Evaluating nutritional status at the start of treatment can improve survival rates. **Purpose:** This study investigated the correlation between the hemoglobin, albumin, lymphocyte count, and platelet (HALP) score and prognosis of patients with metastatic thyroid cancer undergoing first-line TKI therapy. **Methods:** We retrospectively analyzed data from 44 patients between January 2010 and June 2024. The primary outcomes evaluated in the study were time to treatment failure (TTF) and overall survival (OS); HALP scores were categorized as low (≤29.21) and high (>29.21) based on receiver operating characteristic analysis. **Results:** The 1-year survival rate was significantly lower in the low HALP score group compared to the high HALP score group (50% vs. 96.3%). Multivariate Cox regression analysis revealed that low HALP scores, elevated leukocyte counts, and lymphopenia were independent predictors of shorter TTF (HR = 0.272, *p* = 0.011) and OS (HR = 0.208, *p* = 0.028). **Conclusions:** The results obtained in the present study demonstrate that the HALP score has prognostic significance for patients with metastatic thyroid cancer who are undergoing first-line TKI treatment. In metastatic thyroid cancer patients, interventions focused on improving nutritional status at the start, during initiation, and throughout the TKI treatment may enhance treatment effectiveness. However, further prospective studies involving larger patient cohorts are necessary to validate our results.

## 1. Introduction

Thyroid cancer is the most common type of endocrine cancer [1]. Its incidence has been rising recently, and it is more common in women. Nearly all cases present with a thyroid nodule [2]. In the general population, the likelihood of thyroid nodules increases with age, reaching about 60%, with 5–15% of these nodules being malignant. The Bethesda system assesses the probability of malignancy after a fine-needle aspiration biopsy, while a definitive diagnosis is confirmed through surgical resection [3,4,5]. The Bethesda categories 3 and 4 comprise indeterminate thyroid nodules (ITNs), which pose significant diagnostic challenges. Recent advancements in understanding driver mutations and signaling pathways have significantly enhanced diagnostics, particularly for ITNs [6].

Differentiated thyroid cancers (DTCs) represent 95% of thyroid cancer cases and arise [7,8,9,10,11] from follicular cells. They typically respond to radioactive iodine (RAI) treatment. For small tumors, surgery or a combination of RAI and levothyroxine therapy is usually adequate. However, distant metastasis occurs in about 25% of cases, and most metastatic patients are resistant to RAI treatment [7,12]. Medullary thyroid cancer (MTC) constitutes approximately 2% of cases and originates from parafollicular C cells. About 80% of cases are sporadic, while hereditary forms are linked to multiple endocrine neoplasia (MEN) type 2 [13,14,15]. Hereditary forms are associated with genomic mutations in the RET proto-oncogene and exhibit autosomal dominant inheritance. Both RET and RAS mutations have been identified in sporadic cases [16,17].

Tyrosine kinase inhibitors (TKIs) inhibit cell proliferation and neoangiogenesis pathways [18]. TKIs demonstrate high efficacy against unresectable, advanced-stage DTC and MTC [7,19]. Sorafenib and Lenvanitinib are approved in RAI-refractory (RAI-R) locally advanced and metastatic differentiated thyroid cancers [7,9]. The effective combination of dabrafenib and trametinib targets BRAF mutations, providing a promising treatment option for patients with differentiated thyroid cancers while intentionally excluding anaplastic medullary cancer [10]. Vandetanib and cabozantinib are utilized in the systemic treatment of medullary thyroid cancer [20,21]. These drugs significantly contribute to the progression-free survival (PFS) of patients with advanced disease [4,9,14,15,16,17].

Host factors, including the immune response and inflammatory cells, are crucial for cancer treatment outcomes and survival rates [22]. Inflammation scores such as the neutrophil-to-lymphocyte ratio (NLR), platelet-to-lymphocyte ratio (PLR), systemic immune-inflammation index (SII), and systemic inflammatory response index (SIRI) are commonly used parameters that are closely associated with mortality in malignant diseases [23,24,25,26]. Research has shown that inflammatory markers can impact the prognosis of thyroid malignancies [27,28]. Malnutrition is also frequently observed in patients with advanced-stage cancer, negatively impacting prognosis [29]. Evaluate body mass index, weight loss, and food intake, and develop nutritional plans for patients at risk of malnutrition. Daily energy intake should be set at 25–30 kcal/kg/day, while protein intake should be maintained at 1.5 g/kg/day. Nutritional screening should be conducted for patients with advanced thyroid cancer both early on and throughout their treatment [30,31,32].

In cancer patients, the relationship between albumin as a standalone indicator of malnutrition and prognosis and the association between various malnutrition indices and prognosis have been demonstrated in the literature [33,34]. Prognostic nutritional index (PNI) and hemoglobin, albumin, lymphocyte, and platelet (HALP) scores are among the most notable of these scoring systems [35,36]. The association of the HALP score with prognosis has also been demonstrated in various malignancies, including gastric, renal, bladder, small-cell lung, and prostate cancers [36,37,38,39].

The current study aimed to assess the nutritional and inflammatory markers at the initiation of TKI treatment in patients diagnosed with metastatic thyroid cancer and their correlation with disease prognosis. As far as we know, this is the initial study in the literature to evaluate the HALP score in this cohort of patients.

## 2. Materials and Methods

### 2.1. Study Design

**Figure 1:**
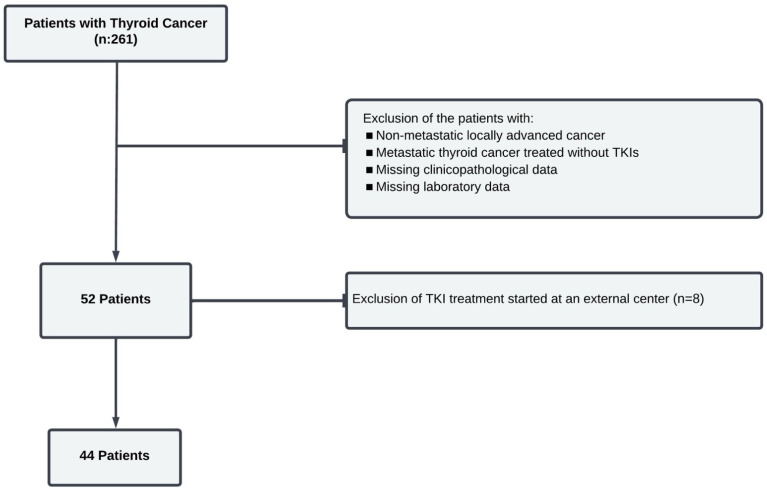
Flowchart of the study.

Eighty-three patients aged 18 years and older, diagnosed with metastatic thyroid cancer, were followed-up at the Bursa Uludag University Endocrinology Department from January 2010 to June 2024. Patients with a diagnosis of metastatic thyroid cancer who did not use tyrosine kinase inhibitors and who started first-line treatment at an external center were excluded from the study. The patient flowchart is illustrated in Figure 1.

### 2.2. Clinical Assesment

Demographic data, clinicopathological features, laboratory parameters, imaging results, pathology findings, sites of metastasis, TKI treatment information, response status, and patient survival outcomes were retrospectively collected from patient files and the hospital information system. Inflammation markers were calculated as follows: The neutrophil-to-lymphocyte ratio (NLR) was determined by dividing the neutrophil count by the lymphocyte count; the platelet–lymphocyte ratio (PLR) was calculated by dividing the platelet count by the lymphocyte count; the systemic immune-inflammation index (SII) was computed as (platelet count × neutrophil count)/lymphocyte count; and the systemic inflammatory response index (SIRI) was determined as (neutrophil count × monocyte count)/lymphocyte count in peripheral blood. To assess nutritional status, the prognostic nutritional index (PNI) was calculated using the formula, 10 × albumin (g/dL) + 0.05 × lymphocyte count (mm^3^). The HALP score was calculated using the formula, (hemoglobin (g/L) × albumin (g/L) × lymphocyte count (×10^9^/L))/platelet count (×10^9^/L). Time to treatment failure (TTF) was defined as the duration from initiating first-line TKI therapy to death or switching to second-line TKI therapy due to disease progression. Tumor progression was assessed using computed tomography or magnetic resonance imaging, adhering to the Response Evaluation Criteria in Solid Tumors, version 1.1. Furthermore, local metastases that did not necessitate a change in TKI treatment were not classified as treatment failures. Overall survival was defined as the time from the initiation of first-line TKI treatment until the final visit or death from any cause.

### 2.3. Statistical Analysis

The data analysis was performed using SPSS version 29.0 (IBM Corp, Armonk, NY, USA). Categorical variables are summarized as counts and percentages, while continuous variables are reported as means and standard deviations for normal distributions or as medians with minimum and maximum values otherwise. Student’s *t*-test or the Mann–Whitney U test was used for comparisons between two groups, and one-way ANOVA or the Kruskal–Wallis test was used for three or more groups. Categorical variables were analyzed using the chi-square test. The HALP score cut-off was established to estimate mortality. Kaplan–Meier and Cox regression analyses were utilized to evaluate time to treatment failure (TTF) and overall survival (OS), with hazard ratios and 95% confidence intervals calculated for both measures. For the multivariate analysis, Cox regression using the Backward Likelihood Ratio method was performed, incorporating factors that had *p*-values less than 0.2 from the univariate analysis. A *p*-value of less than 0.05 was considered statistically significant.

## 3. Results

**Figure 2:**
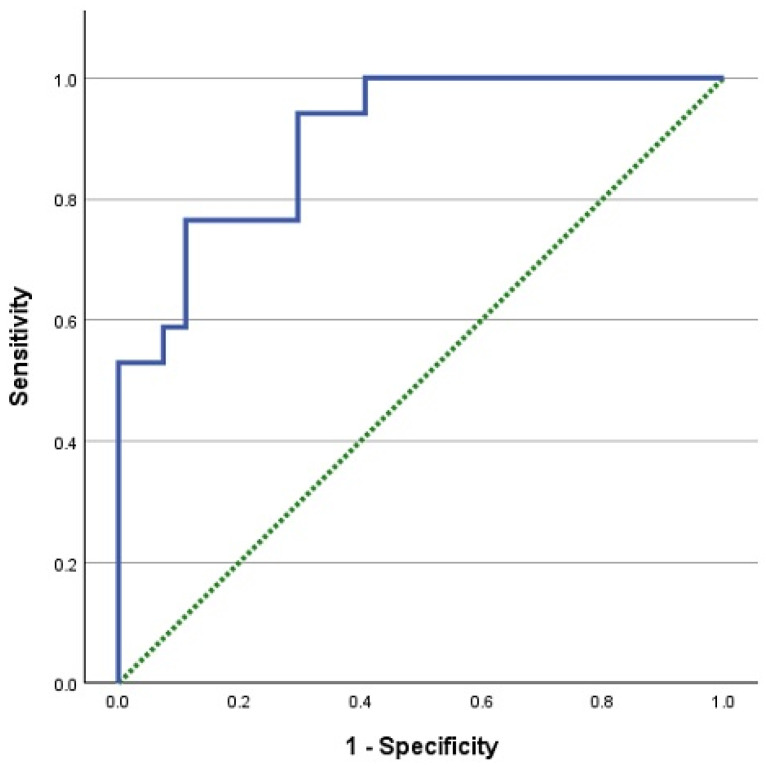
ROC analysis of the HALP score to predict mortality.

The patients’ demographic characteristics and laboratory parameters according to the pathological subtypes of metastatic thyroid cancer are presented in Table 1. Although the frequency of female patients was higher in those with follicular thyroid carcinoma, the male gender ratio was higher in the other two subtypes; however, no statistically significant difference was detected between the groups (*p* = 0.324). Moreover, there was no statistically significant difference between the groups regarding the age at which TKI therapy was initiated and the time from the diagnosis of metastatic thyroid cancer to the start of TKI treatment. No statistically significant differences were found between the groups regarding the rate of visceral metastasis, laboratory parameters at the start of TKI treatment, inflammation, or nutritional scores (*p* > 0.05). According to receiver operating characteristic (ROC) curve analysis, the cut-off value of the HALP score for predicting mortality was 29.21 (AUC: 0.90, sensitivity: 76.5%, specificity: 88.9%, *p* < 0.001). The results of the ROC analysis are depicted in Figure 2. Patients with a HALP score of ≤29.21 were defined as the HALP^low^ group, and those with a HALP score of >29.21 were defined as the HALP^high^ group.

The characteristics of the patients according to their HALP scores are presented in Table 2. No significant differences were observed in demographic data or patient characteristics between the two groups.In addition to the parameters of the HALP score, C-reactive protein (CRP) levels were significantly higher in the HALP^low^ group (*p* = 0.008). No significant differences were observed between the groups regarding other inflammation markers. The PNI score, another indicator of nutritional status, had a median value of 45.6 in the HALP^low^ group and 55.3 in the HALP^high^ group, and this difference was statistically significant (*p* < 0.001).

The findings from the Cox regression analysis for TTF are provided in Table 3. In the univariate analysis, HALP score (low vs. high), total leukocyte count, neutrophil count, and hemoglobin levels were found to be significant. In the multivariate analysis, low HALP scores and leukocyte counts were identified as independent variables associated with shorter TTF (HR: 0.272, HR: 1.185; *p* = 0.011, *p* = 0.005). Other variables were not statistically significant.

The findings from the Cox regression analysis for OS are displayed in Table 4. In the univariate analysis, HALP score (low vs. high), age of TKI initiation, total leukocyte count, neutrophil count, hemoglobin levels, lymphocyte count, serum lactate dehydrogenase levels, serum albumin levels, and PNI score were found to be significant. In the multivariate analysis, low HALP scores, high leukocyte counts, and lymphopenia were identified as independent variables associated with shorter OS (HR: 0.208, HR: 1.178, HR: 0.167; *p* = 0.028, *p* = 0.008, *p* = 0.005). Other variables were not statistically significant.

The results from the collinearity analysis, which investigates the independent variables affecting TTF and OS, are presented in Table 5. The evaluation of the HALP score indicated that there was no multicollinearity among the independent variables.

**Figure 3:**
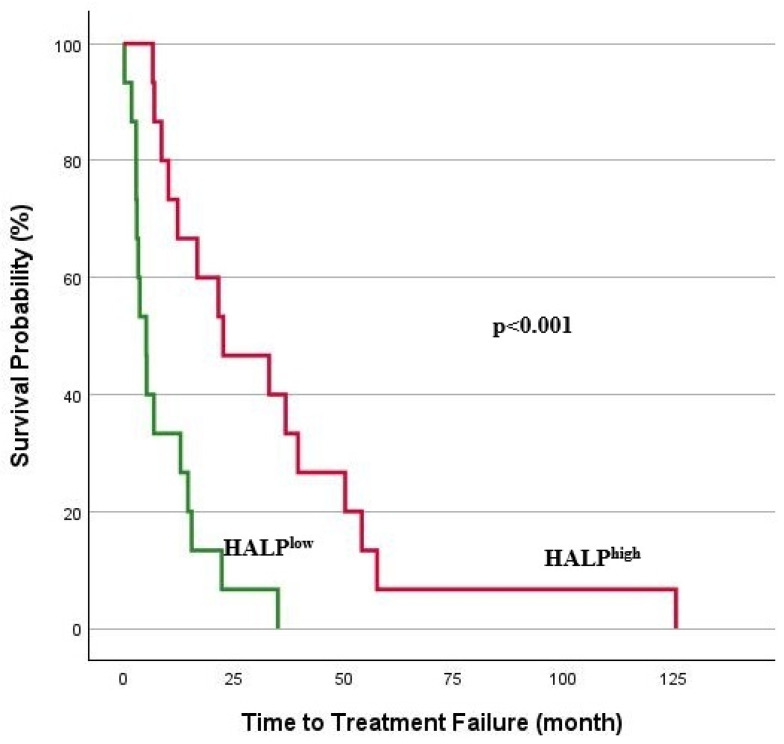
Kaplan–Meier analysis for TTF.

**Figure 4:**
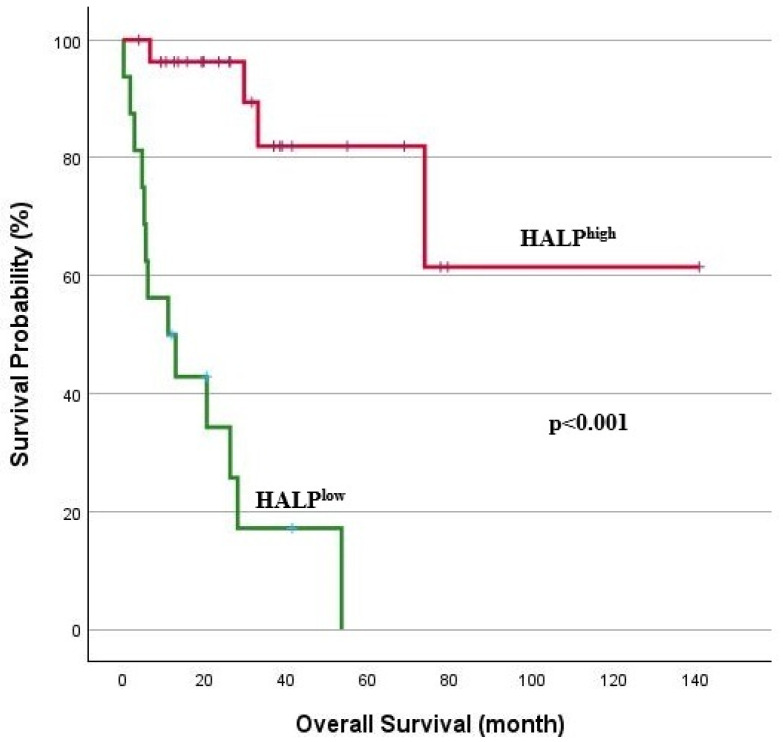
Kaplan–Meier analysis for OS.

The median follow-up period for the study group was 20.2 months (range: 0.3–140.8), and survival analyses for TTF and OS are illustrated in Figure 3 and Figure 4. A log-rank analysis revealed that the median TTF was significantly lower in the HALP^low^ group (5.2 months vs. 22.6 months; *p* < 0.001). Similarly, an OS analysis showed significantly lower overall survival in the HALP^low^ group (*p* < 0.001). In the HALP^low^ group, the median OS was 11.1 months, whereas the median survival was not reached in the HALP^high^ group. The 1-year OS rate was 50% in the HALP^low^ group compared to 96.3% in the HALP^high^ group.

**Figure 5:**
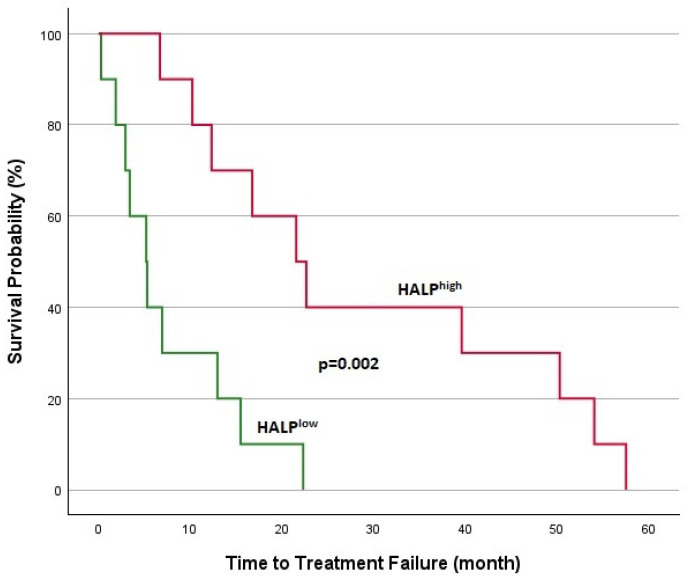
Kaplan–Meier analysis in DTCs for TTF.

**Figure 6:**
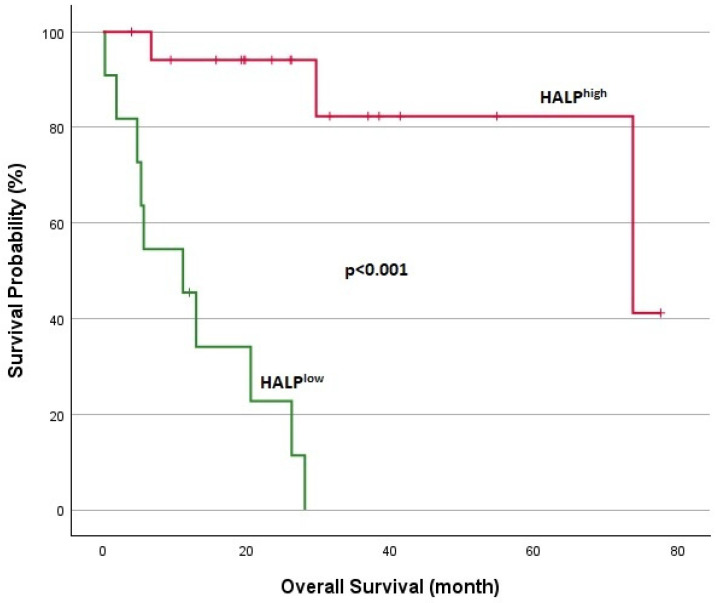
Kaplan–Meier analysis in DTCs for OS.

**Figure 7:**
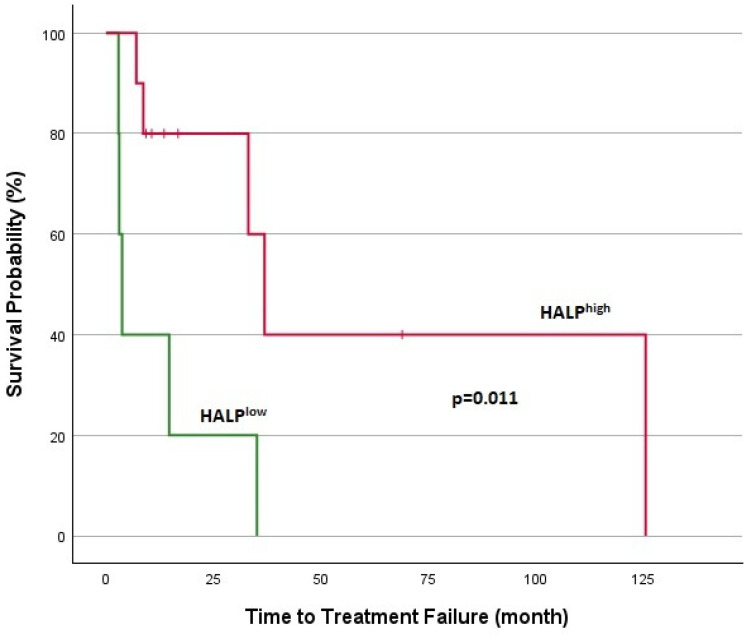
Kaplan–Meier analysis in MTC for TTF.

**Figure 8:**
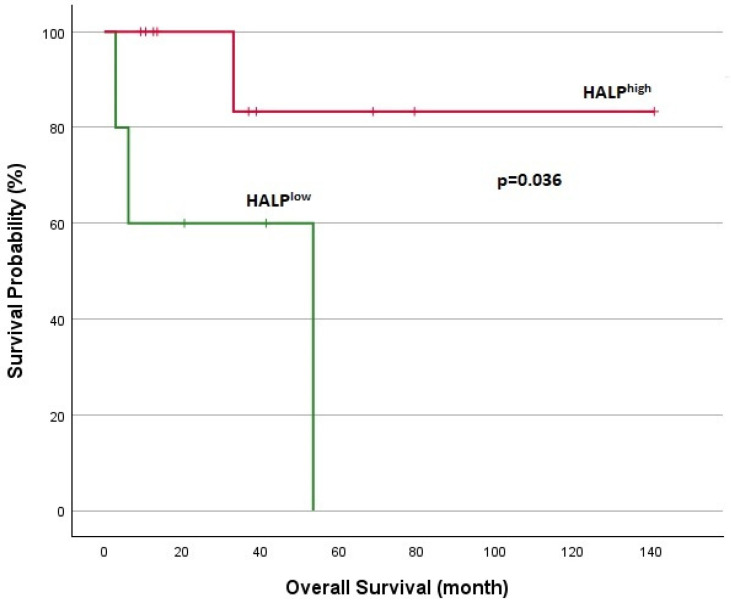
Kaplan–Meier analysis in MTC for OS.

Survival analyses for patients with DTCs are shown in Figure 5 and Figure 6. In DTC patients, the median TTF was 5.2 months for the HALP^low^ group and 21.5 months for the HALP^high^ group, with a statistically significant difference (*p* = 0.002). The 1-year overall survival rate was significantly lower in the HALP^low^ group (44.5% vs. 94.1%; *p* < 0.001). Survival analyses for patients with MTC are shown in Figure 7 and Figure 8. The median TTF was 3.7 months in the HALP^low^ group compared to 36.8 months in the HALP^high^ group (*p* = 0.011). The 1-year OS rate was 60% for the HALP^low^ group, while the HALP^high^ group had no recorded deaths (*p* = 0.036).

## 4. Discussion

The HALP score is an indicator of nutritional, inflammatory, and immune status. Moreover, its relationship with prognosis has been demonstrated in various cancer types, including gastric, breast, esophageal, hepatocellular carcinoma, and lung cancers [36,40,41,42,43]. To the best of our knowledge, this study is the first to evaluate the effect of the HALP score on mortality in metastatic thyroid cancer. In this patient group, when the cut-off value of the HALP score was set at 29.21, TKI response durations and OS were found to be significantly lower in the HALP^low^ group.

The interaction of immune system cells with inflammatory factors in the tumor microenvironment (TME) affects treatment outcomes and prognosis in thyroid cancer [44,45]. Chemokines released from the TME activate inflammatory pathways. The process also results in extracellular matrix (ECM) remodeling. Interleukin-6 (IL-6), interleukin-1 beta (IL-1 beta), and vascular endothelial growth factor (VEGF) are key cytokines involved in these pathways [45,46,47,48]. Increased IL-6 levels in metastatic cancers also contribute to cancer-related cachexia, malnutrition, and sarcopenia [49]. Several studies have investigated the association between inflammation markers and RAI-R, as well as prognosis in thyroid cancer; however, their prognostic significance remains uncertain [50,51,52,53]. Studies suggest that nutritional markers may be prognostic in metastatic RAI-R thyroid cancer. In a study by Dalmiglio et al. involving 42 patients with metastatic thyroid cancer undergoing TKI therapy, those with poor nutritional scores experienced lower rates of progression-free survival and overall survival [54]. Another study by Yamazaki et al. indicated that sarcopenia, a sign of malnutrition, was linked to a poor prognosis in thyroid cancer patients receiving TKI treatment [55]. In our study, the inflammatory markers NLR, PLR, SII, and SIRI had no statistically significant effect on survival outcome. In our study, we found that the HALP score is an independent variable with a prognostic effect on both TTF and OS. The risk of death in patients with a low HALP score is approximately five times higher than in those with a high HALP score.

Lymphopenia, one of the components of the HALP score, has been identified in different cancer types and is known to be associated with poor prognosis [56]. Cytokines such as interferon-γ and TNF-α, secreted by lymphocytes, suppress tumor cell proliferation and invasion while also inducing apoptosis [57,58]. The main components of cancer immune surveillance include CD8-positive (CD8+) cytotoxic T cells, CD4-positive (CD4+) T helper cells, and dendritic cells. When dendritic cells activate effector T cells, they can recognize tumor antigens and trigger a cytotoxic response. Research by Rabold et al. shows that patients with advanced metastatic thyroid cancer have significantly lower levels of CD3+, CD4+, and CD8+ T cells, underscoring the urgent need for strategies to boost T cell activity against cancer [59]. Thus, the possible mechanisms of lymphopenia may include an increase in T cell apoptosis, a simultaneous decrease in the homeostatic proliferation of T cells, reduced thymic output, or the redistribution of T cells within the tumor microenvironment, as described in melanoma and head and neck cancers [60,61]. In a study involving patients with colorectal cancer, disease-free survival and overall OS were found to be lower in the group with lymphopenia at diagnosis [62]. Similarly, it was found that baseline lymphocyte count was associated with immunotherapy efficacy and OS in patients with advanced esophageal cancer overall, and disease-free survival was lower in the group with lymphopenia [63]. Consistent with the literature, lymphopenia was found to be an independent variable for OS in the present study.

Albumin, a negative acute-phase reactant produced in the liver, is an important indicator of nutritional status. Hypoalbuminemia resulting from factors such as malnutrition, systemic inflammation, increased cytokine release, and heightened catabolism leads to a diminished immune response against cancer cells, and its adverse effects on prognosis have been demonstrated in numerous studies [64,65,66]. A study conducted by Wu et al. assessed 208 patients diagnosed with esophageal cancer and discovered that albumin was an independent prognostic indicator [34].

The interaction of platelets with tumor cells through various mechanisms provides protection against TNF-α mediated cytotoxicity, facilitating these cells’ evasion of the immune response and contributing to metastasis [67,68,69]. Similarly, the relationship between hemoglobin levels, survival, and tumor progression is well established. Inflammation and suppression of erythropoiesis resulting from pro-inflammatory cytokines such as IL-6 are leading mechanisms of anemia in cancer patients. Anemia also contributes to tumor hypoxia and treatment resistance [70,71,72,73]. Considering all these mechanisms, the HALP score is a reliable indicator of immunonutritional status. Different cut-off values for the HALP score have been defined across various malignancies [34,36,74]. In a study by Chen et al. involving 1332 gastric cancer patients, the 3-year survival rate was 59.7% for patients with a low HALP score, compared to 74.7% for those with a high HALP score [36]. Similarly, a study by Hu et al. examined 834 esophageal cancer patients and another by Guo et al. (2019) assessed 82 metastatic prostate cancer patients, both demonstrating that patients with a low HALP score had a worse prognosis [34,74]. In the present study, 1-year survival rates were 50% in the HALP^low^ group and 96.3% in the HALP^high^ group.

Research indicates that neutrophils promote cancer cell invasion, proliferation, and metastasis, while also aiding cancer cells in evading immune surveillance [75]. Secondary tissue damage due to malignancy-associated inflammation leads to increased myeloid growth factors and the development of neutrophilia triggered by cytokine release [76]. Tumor-related leukocytosis caused by the release of cytokines such as granulocyte colony-stimulating factor (g-csf), granulocyte-macrophage colony-stimulating factor (gm-CSF), IL-1a,b, IL-3, IL-6, and TNF-α and its prognostic effect have been described in various cancers, including thyroid cancers [77]. In the present study, total leukocyte count, along with HALP score and lymphopenia, yielded statistically significant results for both TTF and OS in multivariate analysis.

Nutritional status assessment and intervention reduces disease-related symptoms and improves prognosis in advanced thyroid cancer treatment. It is also known to positively impact quality of life and OS. Patients should be screened for nutritional status, and enteral or parenteral nutrition support should be considered when necessary [78]. Nutritional interventions can enhance overall survival in thyroid cancer patients undergoing TKI treatment and in various types of malignancies [78,79,80,81]. The guidelines from the European Society of Clinical Nutrition and Metabolism (ESPEN) recommend assessing nutritional status based on several factors. These include body mass index (BMI), weight loss, body composition, loss of muscle mass and/or subcutaneous adipose tissue, inflammatory markers, and eating habits [31,79,82]. Nutrition counseling should be offered alongside dietary regulation [31,82,83]. In a study by Chen et al., thyroid cancer patients who received nutritional counseling showed a significant improvement in quality of life, nutritional screening scale scores, and compliance compared to those who did not receive such counseling [84]. In our patient group, we provided a high-protein diet as a nutritional intervention and enteral/parenteral nutritional support when necessary. However, we have limited information on the integrated use of global nutrition assessment scales and the impact of nutrition support interventions on prognosis.

The limitations of our study include its retrospective design, the use of a limited number of patients from a single center, the absence of a validation cohort, limited follow-up, and an incomplete evaluation of confounding nutritional factors. Furthermore, the substantial number of patients receiving statin therapy, along with the instances of incomplete lipid profile data at the commencement of TKI treatment, posed significant challenges to the accuracy of lipid profile-based nutritional assessments.

## 5. Conclusions

Our research indicates that the HALP score is an important prognostic marker for patients with advanced metastatic thyroid cancer. The HALP score, calculated using simple and cost-effective parameters, serves as a valuable tool for assessing patients’ nutritional status before TKI treatment. Early nutritional support for those with low scores can significantly improve survival outcomes. To validate these findings, it is essential to conduct multicenter studies involving large cohorts of patients. These studies should focus on the dynamic monitoring of nutritional parameters over time, allowing for a comprehensive assessment of the impact of nutrition on treatment outcomes.

## Figures and Tables

**Table 1:** General patient characteristics (n:44).

	Papillary Carcinoma	Follicular Carcinoma	Medullary Carcinoma	*p*-Value
	n:19	n:10	n:15	
**Gender, n *(%)***				
Male	10 (52.7)	4 (36.4)	8 (57.1)	0.324 ^a^
Female	9 (47.3)	7 (63.6)	6 (42.9)	
**Age of TKI initiation *(mean ± SD)***	59.4 (±9.6)	60.4 (±14.4)	58.5 (±9.9)	0.980 ^b^
**Time from diagnosis to TKI initiation, month *(median)***	38.6 (7.7–199.4)	33.7 (2.2–185.1)	11.5 (4.7–164.6)	0.158 ^c^
**Visceral metastasis *(%)***				
No	3 (16)	2 (20)	6 (40)	0.238 ^a^
Yes	16 (84)	8 (80)	9 (60)	
**Hemoglobin, g/dL *(mean ± SD)***	12.7 (±1.86)	12.6 (±1.90)	13.8 (±1.29)	0.283 ^b^
**Albumin, g/L *(median)***	45 (29–51)	40 (32–51)	43 (31–46)	0.445 ^c^
**WBC, 10^9^/L *(median)***	7.9 (4.2–14)	5.9 (3.9–32.9)	6.4 (5.1–17)	0.156 ^c^
**Neutrophils, 10^9^/L *(median)***	5.3 (3–13.2)	3.7 (2.8–31)	4.1 (2.5–15.7)	0.177 ^c^
**Lymphocytes, 10^9^/L *(median)***	1.8 (0.2–2.6)	1.1 (0.4–2.5)	1.5 (0.9–7.4)	0.172 ^c^
**Platelets, 10^9^/L *(median)***	255 (128–542)	209 (69–318)	227 (143–305)	0.080 ^c^
**LDH ^1^, U/L *(median)***	229 (181–489)	245 (199–722)	242 (160–782)	0.899 ^c^
**CRP ^2^, mg/L *(median)***	6.5 (2–107)	6.3 (2–56)	2 (1–169)	0.109 ^c^
**Total cholesterol ^3^, *(%)***				
**<200 mg/d** **L**	5 (50)	4 (80)	5 (55.6)	0.667 ^a^
**≥200 mg/d** **L**	5 (50)	1 (20)	4 (44.4)	
**LDL cholesterol ^3^, *(%)***				
**<130 mg/d** **L**	5 (50)	5 (100)	7 (77.8)	0.133 ^a^
**≥130 mg/d** **L**	5 (50)	0	2 (22.2)	
**Triglyceride ^3^, *(%)***				
**<150 mg/d** **L**	4 (40)	5 (100)	8 (88.9)	**0.029 ^a^**
**≥150 mg/d** **L**	6 (60)	0	1 (11.1)	
**HDL cholesterol ^3^, *(%)***				
**<40 (male) or <50 (female)**	9 (90)	5 (100)	3 (33.3)	**0.008 ^a^**
**≥40 (male) or ≥50 (female)**	1 (10)	0	6 (66.7)	
**PNI, *(median)***	54.1 (30.4–59.8)	45.3 (36.8–60.1)	49.3 (35.5–79)	0.282 ^c^
**NLR, *(median)***	3.1 (0.7–48.9)	2.27 (1.2–4.6)	2.9 (1.8–34.3)	0.582 ^c^
**PLR, *(median)***	140.8 (23.7–1388.9)	158.1 (100.9–202.3)	142.8 (102.1–357.3)	0.868 ^c^
**SII, *(median)***	696 (124.3–18,333.3)	636.8 (359.1–1159.9)	701.7 (294.2–10,919.2)	0.953 ^c^
**SIRI, *(median)***	1.6 (0.2–19.1)	1.1 (0.5–3.7)	1.7 (0.7–37.8)	0.362 ^c^
**HALP score *(median)***	40.1 (2.3–72.3)	29.6 (13.6–57.8)	42.2 (23.7–269.5)	0.454 ^c^
**First-line TKI *(%)***				
Sorafenib	19 (100)	10 (100)	1 (6.7)	**<0.001 ^a^**
Cabozantinib	0	0	6 (40)	
Vandetanib	0	0	8 (53.3)	

^a^: Chi-square, ^b^: One-way analysis of variance, ^c^: Kruskal–Wallis, TKI: Tyrosine kinase inhibitor, WBC: Total leukocyte count, LDH: Lactate dehydrogenase, CRP: C-reactive protein, PNI: Prognostic nutritional index, NLR: Neutrophil–lymphocyte ratio, PLR: Platelet-to-lymphocyte ratio, SII: Systemic inflammatory index, SIRI: Systemic inflammation response index, LDL: Low-density lipoprotein, HDL: High-density lipoprotein; ^1^: Evaluated in 32 patients; ^2^: Evaluated in 41 patients; ^3^: Evaluated in 24 patients, Bold values: Statistical significant.

**Table 2:** Patient characteristics according to HALP score (n: 44).

	HALP^low^ n *(%)*	HALP^high^ n *(%)*	*p*-Value
**Gender, n *(%)***			
Male	7 (43.8)	15 (53.6)	0.75 ^a^
Female	9 (56.3)	13 (46.4)	
**Age of TKI initiation, *(mean ± SD)***	62.4 (±10.2)	57.7 (±11)	0.165 ^c^
**Time from diagnosis to TKI initiation, month *(median)***	29.1 (2.2–162.8)	34.8 (4–199.4)	0.510 ^b^
**Visceral metastasis, *n(%)***			
No	4 (25)	7 (25)	>0.999 ^a^
Yes	12 (75)	21 (75)	
**Hemoglobin, g/dL *(mean ± SD)***	11.8 (±1.5)	13.7 (±1.6)	**<0.001 ^c^**
**Albumin, g/L *(median)***	39 (29–45)	45.5 (35–51)	**<0.001 ^b^**
**WBC, 10^9^/L *(median)***	7.1 (4.2–32.9)	7.3 (3.9–13.2)	0.600 ^b^
**Neutrophils, 10^9^/L *(median)***	5.1 (3–31)	4.4 (2.5–7.6)	0.088 ^b^
**Lymphocytes, 10^9^/L *(median)***	1 (0.2–2)	1.9 (0.4–7.4)	**<0.001 ^b^**
**Platelets, 10^9^/L *(median)***	215.5 (128–542)	236.5 (69–308)	0.961 ^b^
**LDH ^1^, U/L *(median)***	260 (175–782)	216.5 (160–489)	0.138 ^b^
**CRP ^2^, mg/L *(median)***	11.4 (2–107)	2.7 (1–169)	**0.008 ^b^**
**Total cholesterol ^3^, *(%)***			
**<200 mg/dL**	6 (66.7)	8 (53.3)	0.678 ^a^
**≥200 mg/d** **L**	3 (33.3)	7 (46.7)	
**LDL cholesterol ^3^, *(%)***			
**<130 mg/dL**	6 (66.7)	11 (73.3)	>0.999 ^a^
**≥130 mg/d** **L**	3 (33.3)	4 (26.7)	
**Triglyceride ^3^, *(%)***			
**<150 mg/dL**	7 (77.8)	10 (66.7)	0.669 ^a^
**≥150 mg/d** **L**	2 (22.2)	5 (33.3)	
**HDL cholesterol ^3^, *(%)***			
**<40 (male) or <50 (female)**	7 (77.8)	10 (66.7)	0.669 ^a^
**≥40 (male) or ≥50 (female)**	2 (22.2)	5 (33.3)	
**PNI, *(median)***	45.6 (30.4–53.9)	55.3 (40.5–79)	**<0.001 ^b^**
**NLR, *(median)***	3.1 (0.71–48.9)	2.7 (1.1–34.3)	0.373 ^b^
**PLR, *(median)***	156.3 (23.7–1388.9)	142.6 (64.2–713.6)	0.770 ^b^
**SII, *(median)***	636 (124.3–18,333.3)	698.9 (187.5–10,919.1)	0.714 ^b^
**SIRI, *(median)***	1.9 (0.21–19.07)	1.6 (0.47–37.8)	0.400 ^b^

^a^: Chi-square, ^b^: Mann–Whitney U, ^c^: Independent samples T-Test, TKI: Tyrosine kinase inhibitor, WBC: Total leukocyte count, LDH: Lactate dehydrogenase, CRP: C-reactive protein, PNI: Prognostic nutritional index, NLR: Neutrophil–lymphocyte ratio, PLR: Platelet-to-lymphocyte ratio, SII: Systemic inflammatory index, SIRI: Systemic inflammation response index, LDL: Low-density lipoprotein, HDL: High-density lipoprotein; ^1^: Evaluated in 32 patients; ^2^: Evaluated in 41 patients; ^3^: Evaluated in 24 patients, Bold values: Statistical significant.

**Table 3:** Univariate and multivariate Cox regression analysis for time from first-line TKI initiation to treatment failure (TTF).

Factor	Univariate Analysis				Multivariate Analysis			
	HR	95% CI		*p*-Value	HR	95% CI		*p*-Value
		Lower	Upper			Lower	Upper	
**HALP (low [RC] vs. high)**	**0.236**	**0.100**	**0.556**	**<0.001**	0.272	0.100	0.741	**0.011**
**Age of TKI initiation**	1.023	0.985	1.063	0.242				
**Gender**	0.904	0.428	1.909	0.792				
**Diagnosis subgroup**	0.980	0.654	1.469	0.923				
**Visceral metastasis**	0.833	0.349	1.991	0.682				
**WBC**	1.213	1.075	1.369	**0.002**	1.185	1.052	1.335	**0.005**
**Neutrophil**	1.227	1.087	1.384	**<0.001**				
**Monocyte**	3.156	0.488	20.396	0.227				
**Hemoglobin**	0.771	0.632	0.942	**0.011**				
**Lymphocyte**	0.890	0.528	1.501	0.662				
**Platelet**	1.002	0.997	1.008	0.438				
**LDH ^1^**	1.003	1.000	1.005	**0.089**				
**Albumin**	0.610	0.280	1.327	0.213				
**CRP ^2^**	0.986	0.970	1.003	**0.103**				
**Time from onset of metastatic disease to TKI initiation**	0.978	0.955	1.002	**0.074**				
**Total cholesterol ^3^ (high [RC] vs. low)**	0.614	0.230	1.639	0.330				
**LDL cholesterol ^3^ (high [RC] vs. low)**	0.645	0.234	1.773	0.395				
**Trigliyceride ^3^ (high [RC] vs. low)**	1.302	0.448	3.789	0.628				
**HDL cholesterol ^3^ (high [RC] vs. low)**	1.439	0.478	4.333	0.518				
**PNI, *(median)***	0.970	0.918	1.025	0.277				
**NLR, *(median)***	1.025	0.985	1.066	0.231				
**PLR, *(median)***	1.001	0.999	1.003	0.286				
**SII, *(median)***	1.000	1.000	1.000	0.247				
**SIRI, *(median)***	1.028	0.979	1.081	0.268				

RC: Reference category, HR: Hazard ratio, CI: Confidential interval, TKI: Tyrosine kinase inhibitor, WBC: Total leukocyte count, LDH: Lactate dehydrogenase, CRP: C-reactive protein, PNI: Prognostic nutritional index, NLR: Neutrophil–lymphocyte ratio, PLR: Platelet-to-lymphocyte ratio, SII: Systemic inflammatory index, SIRI: Systemic inflammation response index, LDL: Low-density lipoprotein, HDL: High-density lipoprotein; ^1^: Evaluated in 32 patients; ^2^: Evaluated in 41 patients; ^3^: Evaluated in 24 patients, Bold values: Statistical significant.

**Table 4:** Univariate and multivariate Cox regression analysis for overall survival (OS).

Factor	Univariate Analysis				Multivariate Analysis			
	HR	95% CI		*p*-Value	HR	95% CI		*p*-Value
		Lower	Upper			Lower	Upper	
**HALP (low [RC] vs high)**	**0.071**	**0.020**	**0.253**	**<0.001**	0.208	0.052	0.842	**0.028**
**Age of TKI initiation**	1.066	1.007	1.129	**0.029**				
**Gender**	1.248	0.481	3.241	0.649				
**Diagnosis subgroup**	0.690	0.396	1.201	0.189				
**Visceral metastasis**	2.252	0.508	9.988	0.285				
**WBC**	1.175	1.047	1.319	**0.006**	1.178	1.043	1.330	**0.008**
**Neutrophil**	1.271	1.111	1.455	**<0.001**				
**Monocyte**	0.924	0.065	13.081	0.953				
**Hemoglobin**	0.661	0.507	0.861	**0.002**				
**Lymphocyte**	0.137	0.050	0.372	**<0.001**	0.167	0.048	0.579	**0.005**
**Platelet**	1.003	0.996	1.010	0.364				
**LDH ^1^**	1.004	1.000	1.007	**0.024**				
**Albumin**	0.155	0.054	0.442	**<0.001**				
**CRP ^2^**	0.997	0.980	1.013	0.684				
**Time from onset of metastatic disease to TKI initiation**	0.992	0.963	1.022	0.589				
**Total cholesterol ^3^ (high [RC] vs. low)**	1.285	0.385	4.292	0.683				
**LDL cholesterol ^3^ (high [RC] vs. low)**	1.039	0.298	3.621	0.952				
**Triglyceride ^3^ (high [RC] vs. low)**	1.052	0.278	3.983	0.941				
**HDL cholesterol ^3^ (high [RC] vs. low)**	0.264	0.034	2.078	0.206				
**PNI, *(median)***	0.841	0.772	0.915	**<0.001**				
**NLR, *(median)***	1.004	0.957	1.053	0.866				
**PLR, *(median)***	1.001	0.999	1.003	0.538				
**SII, *(median)***	1.000	1.000	1.000	0.950				
**SIRI, *(median)***	0.979	0.906	1.058	0.592				

RC: Reference category, HR: Hazard ratio, CI: Confidential interval, TKI: Tyrosine kinase inhibitor, WBC: Total leukocyte count, LDH: Lactate dehydrogenase, CRP: C-reactive protein, PNI: Prognostic nutritional index, NLR: Neutrophil–lymphocyte ratio, PLR: Platelet-to-lymphocyte ratio, SII: Systemic inflammatory index, SIRI: Systemic inflammation response index, LDL: Low-density lipoprotein, HDL: High-density lipoprotein; ^1^: Evaluated in 32 patients; ^2^: Evaluated in 41 patients; ^3^: Evaluated in 24 patients, Bold values: Statistical significant.

**Table 5:** Collinearity analysis between independent variables affecting survival outcomes.

	Condition Index	Tolerance	VIF
Constant			
WBC	3.285	0.994	1.006
Lymphocyte	5.007	0.994	1.006

WBC: Total leukocyte count; VIF: Variance Inflation Factor. Since the HALP score is analyzed as a dependent variable, the constant line is used, and the Condition Index, Tolerance, and VIF values are not calculated.

## Data Availability

The datasets used and/or analyzed during the current study are available from the corresponding author upon reasonable request.
